# Evaluation of the photosynthetic response of *Ginkgo biloba* as an urban tree to air pollution, soil salinity, and excess humidity

**DOI:** 10.3389/fpls.2026.1746328

**Published:** 2026-02-11

**Authors:** Takumi Matsuura, Souma Okugawa, Eri Yamakita, Takashi Kiyomizu, Yuuri Tsutsui, Atsushi Kume, Yuko T. Hanba

**Affiliations:** 1 Faculty of Applied Biology, Kyoto Institute of Technology, Kyoto, Japan; 2 Faculty of Agriculture, Kyushu University, Fukuoka, Japan

**Keywords:** *Rhododendron × pulchrum*, hydraulic conductance, mesophyll anatomy, nitrogen dioxide, VPD, stomatal conductance, urban

## Abstract

**Introduction:**

Although the use of *G. biloba* as a roadside tree has been slightly declined in Japan, the number of *G. biloba* planted in Europe and other countries has been increasing in recent years because of its high adaptability to diverse environmental stresses. To re-evaluate the value of *G. biloba* as an urban tree, we focused on three environmental stress factors that can be notable in urban environments in Japan: (1) air pollution, (2) soil salinity, and (3) excess humidity. We evaluated the leaf photosynthetic functions of *G. biloba* in response to the above three types of environmental stresses.

**Methods:**

We compared the responses of *G. biloba* to air pollution (Experiment 1) and to soil salinity (Experiment 2) with those of *Rhododendron* × *pulchrum*, the most commonly used roadside shrub in Japan. For experiment 1, we collected branches of *G. biloba* and *R. pulchrum*, which were planted as roadside trees in Kyoto city, in 2014 and 2017 to measure their photosynthetic functions. For experiments 2, we conducted a growth experiment with *G. biloba* and *R. pulchrum* seedlings, supplying 50 mM NaCl for three weeks. Experiment 3, an excess humidity experiment, was conducted only for *G. biloba* seedlings from 2020 to 2022. It involved a two- to three-week growth experiment under excess humidity and recovery conditions.

**Results and discussion:**

*G. biloba* exhibited a smaller decrease in photosynthetic function in response to air pollution and soil salinity stress than *R. pulchrum* did, confirming its robustness to diverse environmental stresses. The low stomatal density, sunken stomata, and thick mesophyll of *G. biloba* contributed to its high tolerance of photosynthetic function to air pollution stress. The low stomatal density, and likely low proportion of xylem conduit, caused photosynthetic function of *G. biloba* to be less sensitive to soil salinity stress. Conversely, *G. biloba* plants grown under excess humidity exhibited reduced leaf mesophyll development, negatively impacting photosynthesis. This suggests that *G. biloba* does not possess high tolerance to excess humidity.

## 1 Introduction

Urban trees have many beneficial functions, such as decorating the landscape (Rehan, 2013), absorbing CO_2_ through photosynthesis (Nowak and Crane, 2002), adsorbing air pollutants (Nowak et al., 2014), cooling through transpiration (Paschalis et al., 2021), and preventing the reflection of sunlight and temperature increases on the road surface by creating shade (Shashua-Bar et al., 2011). Furthermore, urban trees are also helpful to reduce energy consumption in summer (Donovan and Butry, 2009) and have a positive effect on the health of residents (Pataki et al., 2021). The number of tall roadside trees in Japan was approximately 6.3 million in 2023, of which *Ginkgo biloba*, a deciduous broad-leaved tree native to East Asia, was the most widely planted tall tree species for roadside trees (Iizuka and Matsumoto, 2023). One beneficial reason for the *G. biloba* as a roadside tree is that *G. biloba* is highly fire-resistant (Crane, 2019). High fire-resistance is important for roadside trees, because they can sometimes become the source of wildfires or even promote the spread of fires (Molina et al., 2019). Furthermore, *G. biloba* forests in autumn can improve emotional health in urban areas (Wu et al., 2023). However, in recent years, *G. biloba* is becoming less common as a roadside tree in Japan; *G. biloba* grows into a large tree that is difficult to manage, needing a lot of time and effort to clean up the fallen leaves, which are slow to decompose. In fact, in 2023, the composition ratio of *G. biloba* as a tall roadside tree was 8.3%, almost the same as in 2019 (8.2%), while the composition ratio of cherry trees increased from 7.8% in 2019 to 8.3% in 2023 (Iizuka and Matsumoto, 2023). This shows that, in Japan, cherry trees are becoming more popular as a roadside tree than *G. biloba*.

On the other hand, *G. biloba* is recommended as a roadside tree in Europe (Roloff et al., 2018), because *G. biloba* has high adaptability to a wide range of urban stresses, including air pollution as well as soil salinity and drought (Bassuk et al., 2009; Dmuchowski et al., 2019, Dmuchowski et al., 2022). *G. biloba* was introduced to Europe from Japan in about 1730 (Del Tredici, 1991), and since then has become a popular tree for parks and roadside trees because of its decorative qualities (Dmuchowski et al., 2019). Given that *G. biloba* has many beneficial properties, it is necessary to re-evaluate the value of *G. biloba* as an urban tree.

To evaluate the value of *G. biloba* as an urban tree, it is essential to evaluate its photosynthetic function in response to various stresses in the urban environment, because of the following reasons. First, leaf photosynthesis is the most fundamental function that supports the growth and survival of urban trees. Second, the leaf photosynthetic function is extremely sensitive to environmental stress factors, with the sensitivity differing greatly among urban tree species (Hara et al., 2021). The high environmental stress tolerance of *G. biloba* is likely based on the characteristics of its photosynthetic response to environmental stress. Among the important mechanisms involved in the photosynthetic response to environmental stresses of *G. biloba*, there are two possible important factors: 1) stomatal traits and 2) anatomical traits of the leaves and xylem. For example, *G. biloba* leaves have a low stomatal density, sunken stomata from the leaf surface, and a thick mesophyll, which can reduce the harmful effect of air pollutants (Kiyomizu et al., 2019). The morphological variability of *G. biloba* leaves has long been recognized, with the margins of the leaves sometimes forming ovules on female trees and pollen sacs on male trees (Fujii, 1896; Sakisaka, 1958). These highlight the significance of leaf anatomy in the environmental response of *G. biloba*. *G. biloba* is a gymnosperm that has tracheids with small diameters in xylem, in which Gymnosperm trees have overall low hydraulic conductivity compared to angiosperm trees (Gleason et al., 2016). Small conduit diameter in xylem can reduce the uptake of harmful solutes such as NaCl from the soil, possibly preventing the accumulation of harmful solutes that can have a negative impact on photosynthetic functions (Chowdhury et al., 2025). In urban areas, interaction of local urbanization and global climate change elevates urban humid heat stress (Yang et al., 2023). Such change will cause excess humidity for plants, which will become an important environmental stress for urban trees. Although studies on *G. biloba* are limited, excess humidity causes an increase in the stomatal sensitivity of the hybrid aspen (Niglas et al., 2015), and thus, impacts leaf photosynthesis (Oksanen et al., 2018).

In the present study, we conducted three experiments focusing on the following three environmental stress factors for urban trees: (experiment 1) air pollution, (experiment 2) soil salinity, and (experiment 3) excess humidity. In this and the following sections, we will explain the backgrounds of the studies for urban trees concerning air pollution, soil salinity, and excess humidity. First, concerning air pollution, the concentration of air pollutants, such as NO_x_, is high in urban areas, which is largely caused by automobile exhaust gas (Matsumoto et al., 2022; Matsuura et al., 2025). Many previous studies have been conducted on the photosynthetic response of trees to air pollution using fumigation experiments of pollutants, which reported the negative impact of NO_2_ on photosynthesis in hybrid poplar leaves (Karolewski, 2007; Hu et al., 2015) and improved photosynthesis in mulberry leaves (Wang et al., 2019). Given the interaction between air pollutants and the urban environment, field studies in urban areas are essential to understand the photosynthetic response of urban trees. Our previous field studies suggest that *G. biloba* is among the less sensitive trees to air pollutants (Kiyomizu et al., 2019; Matsumoto et al., 2022; Matsuura et al., 2025). In the first experiment, we hypothesized that the photosynthetic functions of *G. biloba* have a high tolerance to air pollution, which is affected by its stomatal and leaf anatomical traits. To test this hypothesis, we compared the photosynthetic response of *G. biloba* to air pollution with that of *Rhododendron* × *pulchrum* Sweet ‘Oomurasaki’ by re-analyzing our published data obtained in field sites in Kyoto city (Matsuura et al., 2025), adding newly obtained data for stomatal and mesophyll anatomical traits. *Rhododendron* species is the most common shrub tree species in Japan, accounting for 40% of the total shrub roadside tree species (Iizuka and Matsumoto, 2023).

Next, regarding soil salinity stress, the application of de-icing salts for winter road maintenance increases the soil salinity, causing a significant decline in urban trees (Equiza et al., 2017). Furthermore, in recent years in Japan, due to climate change, typhoons have become stronger, resulting in flooding and increasing the soil salinity in coastal areas (Goto et al., 2015). Even in inland areas, there has been significant damage to urban trees caused by salt water being blown up by typhoons (Yamamoto et al., 2008). Therefore, tolerance to soil salinity will become important for evaluating the planting of urban trees in the future in Japan. An increase in soil salinity causes stomata closure, degradation of chlorophyll, and the reduced activity of photosynthetic enzymes including Rubisco, resulting in reduced photosynthesis (Negrão et al., 2017). Although the experimental salt treatment of 150 mM significantly reduced the photosynthetic function of *G. biloba* seedlings (Li et al., 2025), urban tree of *G. biloba* is reported to be less sensitive to soil salinity (Dmuchowski et al., 2019). In the second experiment, we hypothesized that the photosynthetic functions of *G. biloba* have high tolerance to soil salinity, which is affected by stomatal and mesophyll anatomical traits. To test this hypothesis, we conducted growth experiments on the seedlings of *G. biloba* and *R. pulchrum* for three weeks under the application of moderate salinity (50 mM NaCl solution), then compared the photosynthetic response of *G. biloba* to *R. pulchrum*. We used newly obtained data in 2019.

Finally, we focused on excess humidity in the third experiment. Urban trees planted in tree pits with impermeable pavements are prone to water shortage (Cui et al., 2022), and as a result, are susceptible to stresses caused by fluctuations in the atmospheric humidity (Gillner et al., 2017). Furthermore, interactions with atmospheric pollutants, such as ozone, aggravate the effect of changing humidity on urban trees (Wang et al., 2020). The wet-gets-wetter, dry-gets-drier paradigm is expected by global warming (Polson and Hegerl, 2017), in which in the humid region such as Japan, increase in mean precipitation and increase in heavy rainfall days are predicted (Kimoto et al., 2005). Additionally, urban humid heat stress is predicted in urban areas (Yang et al., 2023). Such changes in climate will cause excess humidity stress, and long-term exposure to excess humidity causes reductions in photosynthesis, leaf biomass and area, and the growth rate of the aboveground parts of silver birch and hybrid aspen (Oksanen et al., 2018). As previously described, *G. biloba* can be tolerant to diverse environmental stresses. However, the photosynthetic responses of *G. biloba* to excess humidity have been scarcely studied. In addition, studies on the physiological mechanisms related to photosynthetic response to humidity are lacking for urban trees. In the third experiment, we hypothesized that the photosynthetic functions of *G. biloba* have a high tolerance to changes in the atmospheric humidity, in which stomatal and leaf anatomical traits are key traits. To test this hypothesis, we conducted an experiment in 2019–2022, in which the photosynthetic responses of *G. biloba* to long-term excess humidity for three weeks were investigated. The objective of the study was to elucidate the mechanism of environmental stress tolerance in the photosynthetic function of *G. biloba* from the anatomical characteristics of the stomata and mesophyll, based on the results of experiments 1, 2, and 3.

## 2 Materials and methods

### 2.1 Photosynthesis measurements in experiments 1, 2, and 3

The photosynthesis measurements were conducted under the same conditions in experiments 1, 2, and 3. Fully-expanded mature leaves were used for the measurements. Photosynthesis measurements were performed using a photosynthesis system, LI-6400XT (Li-Cor Biosciences, Lincoln, NE, USA). Measurements were performed at a leaf temperature of 25 °C, a flow rate of 500 ml min^–1^, and PPFD (photosynthetic photon flux density) of 1500 μmol m^–2^ s^–1^. Unless otherwise stated, measurements were taken at VPD 1.0–1.3 kPa. For the estimation of the photosynthetic parameters, maximum Rubisco carboxylation rate (*V*_cmax_) and maximum electron transport rate (*J*_max_), the CO_2_ concentration was changed to 400, 300, 200, 100, 60, 400, 500, 600, 800, 1000, 1500, and 1900 μmol mol^–1^, to obtain *A*/*C*_i_ curves. Curve fitting was performed using R package, “plantecophys” (Duursma, 2015), with the same assumptions about Rubisco specificity factors such as *K*_c_, *K*_o_ and Γ^*^ for *G. biloba* and *R. pulchrum*. The photosynthesis rate and stomatal conductance at a CO_2_ concentration of 400 μmol mol^–1^ were abbreviated as *A*_400_ and *g*_s400_, respectively.

### 2.2 Experiment 1: atmospheric pollution stress

**Figure 1:**
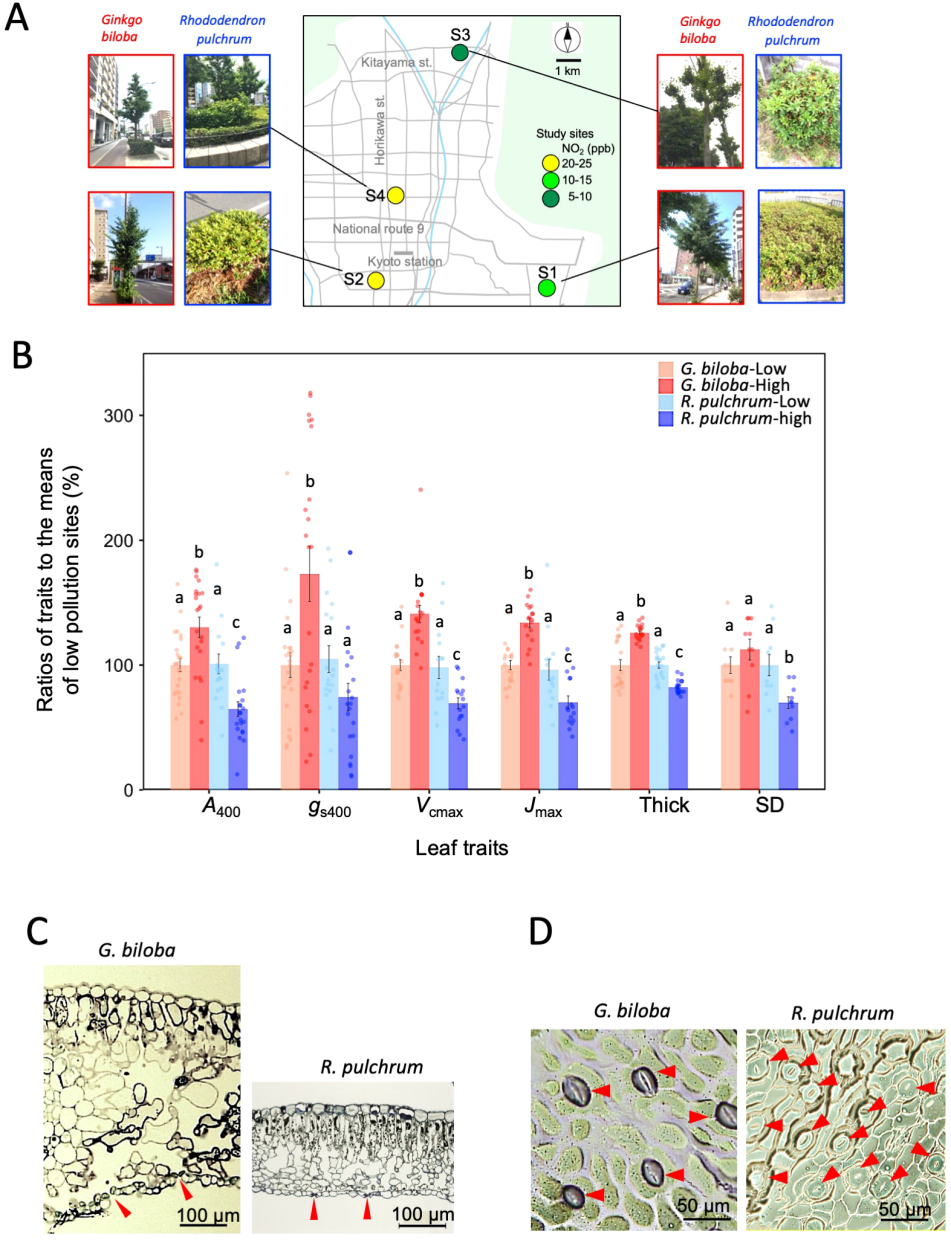
**(A)** Locations of the study sites are shown with tree images for the urban trees, *Ginkgo biloba* and *Rhododendron pulchrum*, at each study site. Concentrations of the atmospheric pollutant NO_2_ at the study sites are shown in color. Branch samples of *G. biloba* and *R. pulchrum* were obtained in 2014 for study sites S1 and S2, and in 2017 for study sites S3 and S4. **(B)** Bar plot for the ratios of the leaf traits of *G. biloba* and *R. pulchrum* at the low and high-pollution sites. The ratios to the mean values of the low-pollution sites are shown. Photosynthetic traits (*n* = 16–24) that is, photosynthesis rate (*A*_400_), stomatal conductance (*g*_s400_), maximum carboxylation rate (*V*_cmax_), maximum electron transport rate (*J*_max_), leaf mesophyll thickness (Thick, *n* = 20) and stomatal density (SD, *n* = 10) are shown. ANOVA and Holm’s *post-hoc* test were used to test for the differences among the four groups: *G. biloba*-low pollution site, *G. biloba*-high pollution site, *R. pulchrum*-low pollution site, and *R. pulchrum*-high pollution site. Different letters indicate statistically significant differences. Error bars indicate SE. Light micrographs of the **(C)** section of the leaves that were chemically fixed and embedded in resin and **(D)** stomata obtained from the secondary replica of the lower sides of the leaves. Red arrows show stomata.

We re-analyzed the data obtained in 2014 and 2017, which was a subset of our published dataset (Matsumoto et al., 2022; Matsuura et al., 2025). In this subset data, branches of *G. biloba* and *R. pulchrum* were collected from the same four study sites in Kyoto city, S1 and S2 in 2014 and S3 and S4 in 2017 (**Figure 1A**). The sampling method was described in the previous study. Briefly, for each study site, the sunlit branches of 3–4 *G. biloba* and *R. pulchrum* trees planted along the roads were collected from July to November. They were brought back to the laboratory and then leaf photosynthesis measurements were performed using the method described in 2.1. In 2014, collection of the branches was conducted 2–4 times per year (3–4 leaves for each sampling time, *n* = 12–16 for each site). In 2017, collection of the branches was conducted in September (two leaves for each tree, *n* = 8 for each site). The anatomical data, stomatal density, stomatal pore length, and mesophyll thickness were obtained by re-analysis of light micrographs of the stomata and leaf sections of 5–6 leaves obtained in 2014. Data on leaf nitrogen content were obtained from Kiyomizu et al. (2019). The anatomical analyses were performed for the leaves in which photosynthesis was measured. For the stomatal density measurements, two randomly-selected areas of 200µm × 200 µm from five images were used, counting the stomata number (*n* = 10). For the measurement of stomatal pore length, four randomly-selected stomata from five images (*n* = 20) were used. Leaf thickness was measured by selecting four randomly-selected locations from five images of leaf section (*n* = 20).

The data of the concentration of atmospheric pollutants were obtained from a public database provided by the Japan National Institute for Environmental Studies (http://www.nies.go.jp/igreen/td_down.html). For the data of the atmospheric pollutants, monthly averaged data from April to November were used. For obtaining the monthly averaged data, daily mean values were used for ozone (O_3_), nitrogen oxide (NO), nitrogen dioxide (NO_2_), and suspended particulate matter (SPM), while for particles that are 2.5 µm or less in diameter (PM_2.5_), the mean of the daily maximum values were used. The meteorological data were obtained from AMeDAS provided by the Japan Meteorological Agency (https://www.data.jma.go.jp/stats/etrn/index.php).

### 2.3 Experiment 2: soil salinity stress

Four seedlings of *G. biloba* and *R. pulchrum*, 30 cm in height, were purchased from a commercial nursery (Kumamoto Greenery Center). The seedlings were grown in pots with a diameter of 13.0 cm and a height of 11.3 cm, filled with a soil mixture of culture soil, Akadama (pumice lava), and perlite at a ratio of 11:11:3. Fertilizer (Hyponex, Hyponex Japan) diluted 500 times with water was applied once a week, 100 ml per pot. The seedlings were grown in a greenhouse at the campus of Kyoto Institute of Technology, with the temperature set at 25 °C. A data logger (HOBO pro v2 U23-002, Onset, Bourne, MA) was placed 1.6 m above the ground in the glasshouse to record temperature and relative humidity every 30 minutes.

We conducted a preliminary experiment using 50, 100, 150 and 200 mM NaCl. We chose 50 mM NaCl because the photosynthesis of *R. pulchrum* decreased to nearly zero at concentrations above 100 mM NaCl. Before the salt treatment (control), the plants were sufficiently watered for three weeks, that is, watered with 150 ml of tap water every other day. For the salt treatment of three weeks, 150 ml of 50 mM NaCl solution was supplied per pot every other day. Images of the whole plant were taken using a digital camera (PowerShot SX 210 IS, Canon, Tokyo and TOUGH TG-5, OLYMPUS, Tokyo).

Fully-expanded undamaged leaves were selected per individual and the photosynthesis rate was measured twice for each leaf using the method described in 2.1 (*n* = 8). To estimate the chlorophyll content of the leaves, we measured the SPAD value, which is an indicator of chlorophyll content, using a chlorophyll meter (SPAD-502Plus, Konica Minolta, Tokyo) for the leaves used for gas exchange measurements (*n* = 4). The SPAD value was measured at six places on each leaf, excluding the leaf veins, and the average value was calculated.

To measure stomatal density, a silicone rubber (KE-14, Shin-Etsu Silicone, Tokyo) and a catalyst (CLC-229, Shin-Etsu Silicone) were mixed and applied to the lower surface of fully-expanded mature undamaged leaves to create a primary replica. A secondary replica of the leaf was made using nail varnish, and then stomata images of the secondary replicas were taken using a digital camera (DP22, Olympus) with a light microscope (BX51, Olympus). Stomatal density and stomatal pore length were measured in four leaves from different individuals before the salt treatment. One image per leaf was used to measure stomatal density (*n* = 4), and five stomata per image were used to measure stomatal pore length (*n* = 20).

To measure the amount of Na in the leaves, the dried leaves were ground, and then 25 ml of distilled water was added per 0.5 g of dried leaves. After centrifugation (14000 rpm) for 15 minutes, 10 ml of the supernatant was filtered through a Stericup-GP (filter pore size 0.22 μm, Merck, German), and then the amount of Na was measured twice (*n* = 8) using an ICP emission spectrometer (ICPE-9820, Shimadzu, Kyoto) at the Center for Environmental Science, Kyoto Institute of Technology.

To evaluate the changes in leaf hydraulic conductance, leaf water potential was measured using a pressure chamber (Model 600 Pressure Chamber Instrument, PMS Instrument Company, Oregon, USA) in four leaves for each species. Leaf water potential at predawn (Ψ_predawn_, 03:00-05:30) and during the day (Ψ_midday_, 11:30-12:30) were measured using one fully-expanded mature undamaged leaf each from four individuals of each tree species. The leaf hydraulic conductivity (K_leaf_, mmol MPa^–1^ m^–2^ s^–1^) was calculated using the following equation (Sperry and Pockman, 1993).


$$K_{leaf}\;=\;\frac{-E}{\Psi_{midday}\;-\;\Psi_{predawn}}$$

where *E* (mmol m^–2^ s^–1^) is the leaf transpiration rate. We assumed that the *E* values at predawn were zero. We obtained *E* values twice at midday using leaf gas exchange measurements with a Li-6400 XT (*n* = 8 for *K*_leaf_). The leaf temperature was 25 °C, the flow rate was 500 ml min^–1^, PPFD was 1500 μmol m^–2^ s^–1^, VPD was 1.0 kPa, and CO_2_ concentration was 400 μmol mol^–1^.

### 2.4 Experiment 3: response to long-term excess humidity

#### 2.4.1 Plant growth

Growth experiments were conducted in 2021 and 2022 for *G. biloba* only. Seedlings of *G. biloba* were purchased from a commercial nursery (Kumamoto Greenery Center). The height of the seedlings was 40–50 cm. The seedlings were transplanted to 4-L nursery containers (17.5 cm diameter, 17 cm height) and filled with mixed soil (culture soil: red clay: pearlite = 11:11:3). The seedlings were grown in a glasshouse at 25 °C for one month and watered sufficiently, and then, six seedlings (*n* = 6) were transferred to a growth chamber (LPH-220/350S, Nihon Medical Instrument Manufacturing, Osaka, Japan) and were grown for 2–3 weeks under “control” conditions (1.5 kPa of VPD, relative humidity of 56%) at a temperature of 25 °C, day/night of 14 h/10 h, and PPFD of 270 µmol m^–2^ s^–1^. Thereafter, they were grown under “excess humidity” conditions (0.5 kPa of VPD, relative humidity of 86%) for about three weeks, and then they were returned to the control condition (“recovery”, 1.6 kPa of VPD, relative humidity of 57%) for about two weeks. During the experiment, the plants were watered three times a week and fertilized once a week with a liquid fertilizer (HYPONeX Japan, Osaka, Japan) diluted 500 times.

In the 2021 experiment, we estimated *V*_cmax_ and *J*_max_ from the *A*-*C*_i_ curves for the control, excess humidity, and recovery conditions for fully-expanded mature leaves (*n* = 4–6, from different individuals) using the instrument and method described in 2.1. The VPD value was set at 1.3 kPa for obtaining the *A*-*C*_i_ curves. In the 2022 experiment, the effects of treatments on leaf anatomy were examined using young, expanding leaves from the top of the shoot apex. These leaves had an area of 18.6 ± 4.3 (mean ± SD) cm^2^, *n* = 6, which was approximately half that of mature leaves, which had a 35.4 ± 14.4 cm^2^, *n* = 12. The leaf area of *G. biloba* increases almost linearly with leaf developmental stage, reaching its maximum approximately 60 days after bud break (Kinoshita et al., 2021). By the end of the recovery treatment, that is, 30–40 days after the start of the excess humidity treatments, the leaf area of the young leaves (34.4 ± 10.8 cm^2^, *n* = 6) was nearly equivalent to that of mature leaves. The response of the stomata to the short-term increase in VPD was determined for five leaves (*n* = 5, from different individuals) using the following method. First, VPD values were set at 0.8 kPa for 2 hours, and then, VPD was increased to a high value, 3.0 kPa, for 30 min. The time-courses of the photosynthesis rate (*A*) and stomatal conductance (*g*_s_) were monitored every 15 seconds during the experiments. First, the mean values of *A* and *g*_s_ were obtained at 0.8 kPa. Then, the changes in *A* and *g*_s_ from 0.8 kPa to 3.0 kPa of VPD were calculated as ratios to the 4-minute averaged value at 0.8 kPa of VPD.

#### 2.4.2 Stomata and leaf anatomical observations

Using the same method as in experiment 2, we made primary and secondary replicas of the abaxial side of the young leaves and obtained images of the secondary replicas with a digital camera (DP22, Olympus) and an optical microscope (BX51, Olympus). We obtained four images from each individual tree for each experiment, in which we counted the stomata of 0.37 mm^2^ areas in the image for three to four individuals (*n* = 12 – 16). Three images of individuals in focus were selected, and eight stomata were chosen from each image to measure their pore length (*n* = 24).

For the analysis of leaf mesophyll anatomy, 5 mm × 2 mm sections of young leaves were obtained. The sections of the leaves were fixed in a 2.5% glutaraldehyde solution in 0.2 M sodium phosphate buffer (pH 7.4). The sections were then dehydrated in 40, 60, 80, and 100% (v/v) ethanol series for 30 min each, and then they were soaked in a solution of ethanol: LR White resin (LR White, Electron Microscopy Sciences, Pennsylvania, USA) = 1:1 for one night. The sections were then soaked with 100% LR White resin solution for 2 hours, and with 100% LR White resin with toluidine blue added for 1 hour, and finally, they were polymerized in an oven at 70 °C for 24 hours. Then, 500-nm thick sections were obtained using an ultramicrotome EM UC6 (Leica Microsystems, Tokyo, Japan) and observed under a light microscope. Images were obtained using a digital camera DP22 (Olympus Corporation, Tokyo) at a magnification of ×1000. The images were analyzed using the software ImageJ (Schneider et al., 2012). We obtained 5–6 section images from different individuals for each experiment, in which two parts of the sections for each image were used (*n* = 9–12). We measured the thickness of the mesophyll and chloroplast surface area facing the intercellular space, *S*_c_, following a previous study (Kiyomizu et al., 2019). To evaluate the development of the mesophyll tissue, we counted the number of mesophyll cell layers in one or two positions in the five images from different individuals (*n* = 9–10 for each treatment). We also measured mean size of three palisade and spongy cells in each position; at the top of the palisade tissue, and in the central part of the spongy tissue for each position, respectively (*n* = 9–10 for each treatment).

### 2.5 Statistical analysis

In experiment 1, that is, the air pollution investigation, we conducted ANOVA and Holm’s *post-hoc* test, using monthly mean data of the pollutants, to compare the air pollutant levels between low- and high-pollution sites for *G. biloba* and *R. pulchrum*. First, we first calculated the average leaf trait values for *G. biloba* and *R. pulchrum* in low-pollution sites 2014 and 2017 separately. Then, we calculated the ratio relative to the low-pollution area for all data from both years. This minimized the impact of differences in climate conditions between the two years. Finally, we pooled the data from both years and performed an ANOVA and a Holm’s *post-hoc* test on the trait ratios for *G. biloba* and *R. pulchrum*.

In experiment 2, that is, 50 mM NaCl treatment, we measured the traits using the same tree for both the control and the NaCl treatment. We calculated the increase in concentration of leaf Na and decrease in *K*_leaf_ for each individual tree after three weeks of 50 mM NaCl treatment. These values were compared between *G. biloba* and *R. pulchrum*, using Welch’s *t*-test. The ratios of the leaf traits of 50 mM-NaCl treatment to those of the controls were calculated for each individual tree. The differences in the leaf traits between *G. biloba* and *R. pulchrum* were tested using Welch’s *t*-test. We first performed ANCOVA for the relationships between leaf traits, considering “species” as a covariate. The effect of species was not statistically significant (p > 0.1) for the relationship between *A*_400_ and *g*_s400_, so we pooled the data for the two species and performed a regression analysis. However, for the relationship between *g*_s400_ and K_leaf_, the species effect was statistically significant (p< 0.05). Therefore, so we performed separate regression analyses for the two species.

In experiment 3, that is the long-term excess humidity experiment for *G. biloba*, the ratios of the leaf traits at excess humidity and recovery were calculated with respect to the traits under the control condition, which is similar to experiment 1. ANOVA and Holm’s *post-hoc* test were used to analyze trait ratios, as well as time-dependent changes in the decrease of *A*_400_ and *g*_s400_.

For all statistical analyses, the outlier values were eliminated using the Kolmogorov–Smirnov test. We performed statistical analysis using R version 4.5.0 and R Commander Version 2.9-5.

## 3 Results and discussion

### 3.1 Experiment 1: atmospheric pollution stress

High atmospheric pollution sites had higher concentrations of NO_2_, NO, and SPM than low atmospheric pollution sites, while PM_2.5_ and O_x_ showed no difference between high and low pollution sites (**Figure 1A**, **Table 1**). Although many previous studies reported negative impacts of O_3_ on the photosynthesis of urban trees (Xu et al., 2015; Gao et al., 2016; Hoshika et al., 2020), the variation in O_x_ in Kyoto city is very small (Matsumoto et al., 2022), in which changes in O_x_ levels are independent of the changes in other air pollutants (Kiyomizu et al., 2019). In Kyoto city, concentrations of air pollutants other than ozone are strongly correlated with traffic volume (Kiyomizu et al., 2019; Matsumoto et al., 2022). It is likely that multiple air pollutants emitted from vehicles collectively affect the photosynthesis of urban trees in Kyoto city. Several studies reported toxic effects of atmospheric pollutants NO_2_ and NO on photosynthesis. Research on *Arabidopsis thaliana* showed that exposure to NO_2_ leads to oxidative stress and functional impairment, which enhances the expression of genes associated with the production and metabolism of harmful reactive oxygen species (Antenozio et al., 2024). In the *Populus* species, exposure to NO_2_ significantly reduces photosynthesis, which is related to partial stomatal closure and changes in chloroplast ultrastructure (Hu et al., 2015). In the present study, the negative effect of atmospheric pollution on the photosynthetic traits *A*_400_*, g*_s400_*, V*_cmax_, and *J*_max_ was significantly different between *Ginkgo biloba* and *Rhododendron pulchrum* (**Figure 1B**; **Supplementary Table S1**). High levels of atmospheric pollutants increased these traits for *G. biloba* but overall decreased them for *R. pulchrum*. This suggests that photosynthesis in *G. biloba* had a high tolerance to atmospheric pollution, which supports our previous studies (Kiyomizu et al., 2019; Matsumoto et al., 2022; Matsuura et al., 2025).

**Table 1:** Concentrations of the atmospheric pollutants nitrogen dioxide (NO_2_), nitrogen monoxide (NO), suspended particle matter (SPM), particles that are 2.5 µm or less in diameter (PM_2.5_), and photochemical oxidants (O_x_) in the low and high pollution sites.

Year	Study sites (pollution level)	NO_2_ (ppb)	NO (ppb)	SPM (g m^–3^)	PM_2.5_ (μg m^–3^)	O_x_ (ppb)
2014						
	S1 (Low)	11.3(0.7)a	2.0(0.5)a	19.1(1.7)ab	31.7(3.9)a	36.6(3.6)a
	S2 (High)	25.1(1.3)b	18.3(2.1)b	23.0(1.8)b	32.9(4.2)a	34.8(3.7)a
2017						
	S3 (Low)	7.0(0.8)c	0.6(0.4)a	13.6(1.5)a	ND	37.5(3.9)a
	S4 (High)	22.9(0.9)b	18.3(2.2)b	21.0(1.1)b	27.3(1.1)a	37.8(3.8)a

Means (SE) are shown for study sites in Kyoto city, in which data were obtained from a public database (http://www.nies.go.jp/igreen/td_down.html). For O_x_ at the high pollution sites, data from the nearest stations to S2 and S4 were used. Annual mean values for 2014 and 2017 were calculated using the monthly maximum of daily averaged values for each month was used. Monthly mean data were used for NO_2_, NO, SPM. For O_x_, the monthly average of daytime one-hour values were used, and for PM_2.5_, the monthly average of the highest daily value for each month was used. The difference among four sites was analyzed using ANOVA and Holm’s *post-hoc* test using monthly data. Different letters indicate a significant difference (p< 0.05).

The high tolerance of photosynthesis to atmospheric pollution in *G. biloba* should be related to the anatomical traits of the leaves that mitigate the harmful effects of air pollutants. First, mesophyll thickness of *G. biloba* was 1.6–2.4-fold compared to *R. pulchrum* (**Figure 1C**, **Table 2**). Furthermore, high atmospheric pollution increased mesophyll thickness of *G. biloba* (**Figure 1B**). A large mesophyll thickness would increase the path length and resistance for the diffusion of air pollutants to the upper mesophyll tissues (Kiyomizu et al., 2019). Second, the stomatal density of *G. biloba* was only 0.27–0.43-fold of *R. pulchrum* (**Figure 1D**, **Table 2**). Low stomatal density and sunken stomata from the leaf lower epidermis in *G. biloba* (**Figure 1D**) would reduce the influx of atmospheric pollutants into the leaves (Kiyomizu et al., 2019). It should be noted that *G. biloba* had larger stomatal pore length, 1.8-fold that of *R. pulchrum* (**Table 2**, **Figure 1D**), which potentially increases the stomatal conductance and thus diffusion of air pollutants into the leaves. There is a strong negative correlation between stomatal density and stomatal size (Franks and Beerling, 2009), so it is not easy to determine which is the more decisive factor for stomatal conductance. In the introgression lines of *Solanum lycopersicum*, stomatal pore size rather than stomatal density is reported to be key for the genetic variation of stomatal conductivity (Fanourakis et al., 2015). In contrast, low stomatal density relates to the low stomatal conductance in *Arabidopsis taliana* (Tulva et al., 2024) and in a variety of rice plant (Caine et al., 2023). In the low pollution site in the present study, *g*_s400_ of *G. biloba* is only 0.6 times that of *R. pulchrum* (**Table 2**). This suggests that, in terms of gas conductivity through the stomata, the effect of lower stomatal density is more pronounced than that of larger stomatal pore size in *G. biloba*.

**Table 2:** Absolute values of mesophyll thickness, stomatal density, stomatal pore length and leaf nitrogen content in the experiment 1.

Species study site (pollution level)	Mesophyll thickness (µm)	Stomatal density (numbers mm^–2^)	Stomatal pore length (µm)	Leaf nitrogen content (g m^–2^)
*Ginkgo biloba*				
S1 (Low)	321(14)a	89(6)a	24.7(0.9)a	2.03(0.14)a
S2 (High)	404(4)b	100(7)a	25.0(0.9)a	2.05(0.15)a
*Rhododendron pulchrum*				
S1 (Low)	204(5)c	332(28)b	13.9(0.5)b	1.12(0.03)b
S2 (High)	168(2)d	232(15)c	13.7(0.4)b	0.83(0.04)b

Sample collections were performed in the year 2014. The means (SE) are shown for mesophyll thickness (*n* = 20), stomatal density (*n* = 10), stomatal pore length (*n* = 20) and leaf-area-based leaf nitrogen content (*n* = 10–16). Leaf nitrogen content data were re-analysis of our previous study (Kiyomizu et al., 2019). ANOVA and Holm’s *post-hoc* test were used to analyze the effects of the treatment and species, where different letters indicate a significant difference (p< 0.05).

Unlike with the photosynthetic traits, for both tree species, pollution levels alter neither the leaf chlorophyll content (SPAD value, **Supplementary Table S1**) nor leaf nitrogen content (**Table 2**). *G. biloba* had significantly higher SPAD and leaf nitrogen content than *R. pulchrum* at both high and low pollution sites (**Supplementary Table S1**; **Table 2**). However, *G. biloba* did not exhibit higher photosynthetic rates than *R. pulchrum* at either S1 or S2 sites (see **Supplementary Table S1**), suggesting that high leaf nitrogen content does not correlate with high photosynthetic rates. Research in Chinese cities reported increased chlorophyll levels in urban areas with elevated NO_x_, suggesting that NO_x_ absorbed by leaves contributes nitrogen for chlorophyll biosynthesis (Yan et al., 2024; Zhang et al., 2024). However, this response was less possible in our study, because no increase in leaf nitrogen content in areas with higher pollution was obtained (**Table 2**).

### 3.2 Experiment 2: salt stress

**Figure 2:**
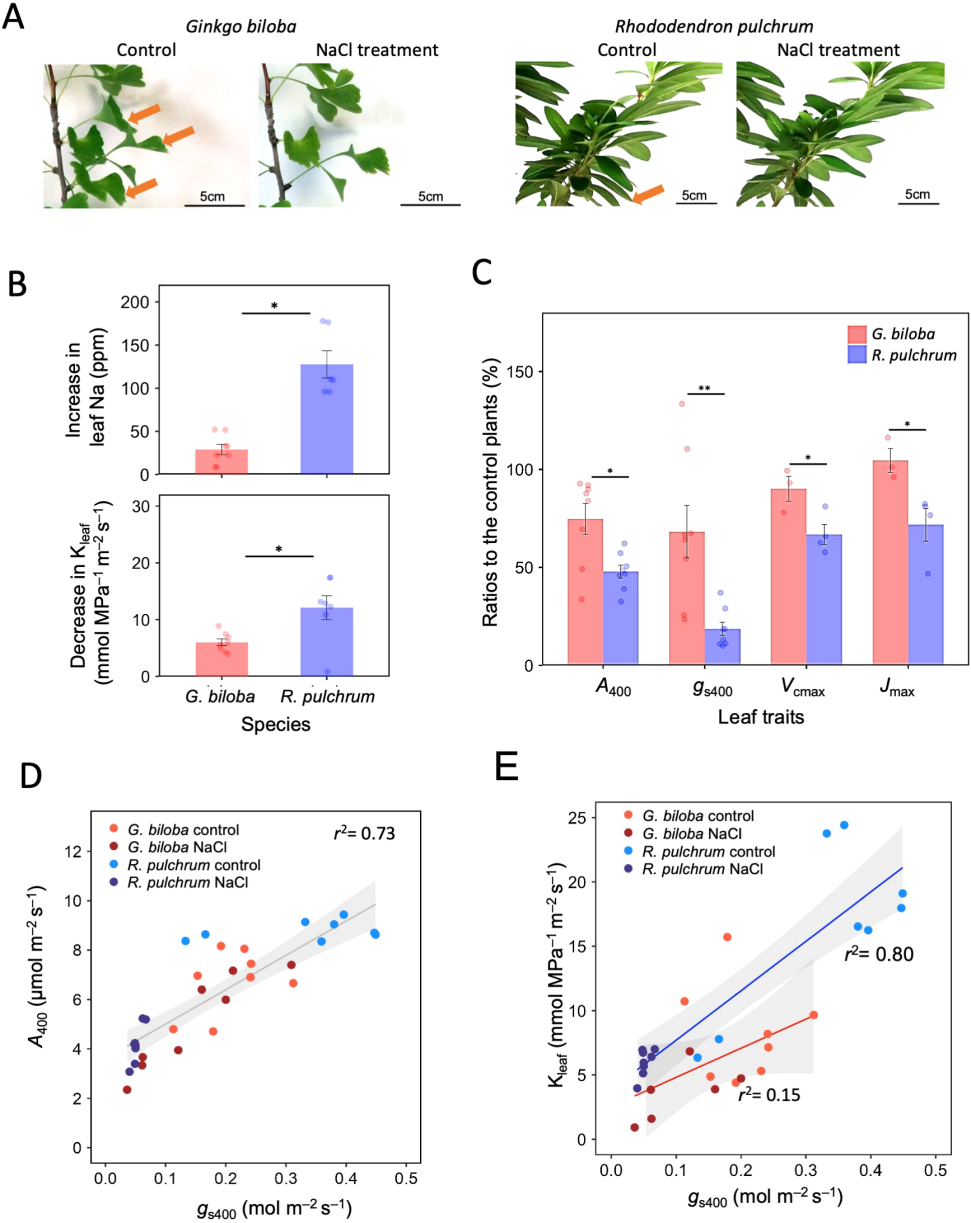
**(A)** Images of the branches for the control and 50 mM NaCl-treated seedlings of *Ginkgo biloba* and *Rhododendron pulchrum.* Orange arrows show leaves that fell after the 50 mM-NaCl-treatment. **(B)** Bar plot for increase in leaf Na content and decrease in leaf hydraulic conductance (K_leaf_) after the 50 mM-NaCl-treatment (*n* = 6–8). **(C)** Bar plot for the ratios of leaf gas exchange traits of (*G*) *biloba* and *R. pulchrum*. Photosynthesis rate (*A*_400_, *n* = 8), stomatal conductance (*g*_s400_, *n* = 8), maximum carboxylation rate (*V*_cmax_, *n* = 4), and maximum electron transport rate (*J*_max_, *n* = 4) are shown. Welch’s t-test was used to test for the differences between species in **(B, C)**. The significance levels are shown as symbols *p < 0.05, **p < 0.01. Error bars indicate SE. **(D)** Relationship between *A*_400_ and *g*_s400_ (*n* = 32) and **(E)** between leaf hydraulic conductance (K_leaf_) and *g*_s400_ (*n* = 14–16) for (*G*) *biloba* and *R. pulchrum*. The solid line shows the regression line, and the 95% confidence interval is shown as a shaded area. The values of *r*^2^ of the regression lines are shown.

A 50 mM NaCl treatment, that is, moderate salinity, resulted in partial leaf falls in both *G. biloba* and *R. pulchrum* (**Figure 2A**), which indicates that 50 mM salt treatment imposed harmful effect on the leaves of both species. On the other hand, accumulation of Na in the leaves after salt treatment was much lower in *G. biloba* than in *R. pulchrum* (**Figure 2B**, **Table 3**). The decrease in K_leaf_ was also lower in *G. biloba* than in *R. pulchrum* (**Figure 2B**, **Table 3**). Accordingly, the degree of the harmful effects of salt treatment on leaf photosynthesis was smaller in *G. biloba* than in *R. pulchrum*; *G. biloba* exhibited a smaller decrease in the photosynthesis rate, 25% reduction in *A*_400_, compared to *R. pulchrum*, which showed a 52% decrease in *A*_400_ (**Figure 2C**).

**Table 3:** Absolute values of leaf Na content, K_leaf,_ and other leaf traits of *Ginkgo biloba* and *Rhododendron pulchrum* subjected to moderate salt stress by applying 50 mM NaCl solution.

Species treatment	Leaf Na content (ppm)	K_leaf_ (mmol m^–2^ s^–1^)	*A*_400_ (µmol m^–2^ s^–1^)	*g*_s400_ (mol m^–2^ s^–1^)	*V*_cmax_ (µmol m^–2^ s^–1^)	*J*_max_ (µmol m^–2^ s^–1^)	Stomatal density (numbers mm^–2^)	Stomatal pore length (μm)
*Ginkgo biloba*								
Control	9.4(0.9)a	8.3(1.3)a	6.71(0.47)a	0.208(0.022)a	34.3(2.8)ab	67.2(5.8)a	56(3)a	19.9(0.9)a
50 mM NaCl	38.3(8.4)b	2.2(1.1)b	5.03(0.68)ab	0.145(0.033)ab	41.5(2.9)b	80.1(5.8)a	ND	ND
*Rhododendron pulchrum*								
Control	2.8(1.0)a	16.5(2.3)c	8.79(0.14)c	0.333(0.043)c	35.3(2.0)a	69.1(3.9)a	189(5)b	12.4(0.2)b
50 mM NaCl	130.5(24.2)c	6.0(0.4)ab	4.19(0.27)d	0.052(0.003)d	26.1(3.0)b	50.2(5.7)a	ND	ND

Means (SE) are shown for leaf Na content (*n* = 6–8), K_leaf_ (*n* = 8), *A*_400_ and *g*_s400_ (*n* = 8), *V*_cmax_ and *J*_max_ (*n* = 4), stomatal density (*n* = 4), and stomatal pore length (*n* = 20). The effects of the treatment and species were analyzed using ANOVA and Holm’s *post-hoc* test, where different letters indicate significant difference (p < 0.05)

What is the physiological mechanism that causes the smaller decline in photosynthesis under salt stress in *G. biloba*? The smaller decline in *g*_s_400_ under salt treatment in *G. biloba* (**Figure 2C**), together with the strong linear correlation between *A*_400_ and *g*_s400_ (**Figure 2D**), suggest that stomatal regulation is key for the different response of photosynthesis to salt stress between *G. biloba* and *R. pulchrum*. Then, why is *g*_s400_ of *G. biloba* less responsive to salt stress than *R. pulchrum*? Although the lower stomatal density and large stomatal pore size of *G. biloba* may impose an opposite effect on stomatal conductance (**Table 3**), as previously explained, in terms of gas conductivity through the stomata, the effect of lower stomatal density should be more pronounced than that of larger stomatal pore size in *G. biloba*. In strawberry cultivars, low stomatal density is correlated with a low sensitivity of stomatal conductance to salt stress (Orsini et al., 2012). Low stomatal density also induces low transpiration, and thus, involves small K_leaf_ as well as low *g*_s_. Such a relationship is shown as a linear correlation between K_leaf_ and *g*_s_ (**Figure 2E**).

Another possible trait that may cause a lower reduction in *A*_400_ under salt treatment in *G. biloba* is low sensitivity of the photosynthetic biochemistry, that is, lower reductions in *V*_cmax_ and *J*_max_ (**Figure 2C**) to salt stress. Salt treatment is reported to reduce *V*_cmax_ and/or *J*_max_ significantly in *G. biloba* and in a thermophilic tree, *Ziziphus* sp*ina-christi*, which is connected to a reduction in the leaf photosynthesis rate (Zait et al., 2019; Li et al., 2025). Then, why are *V*_cmax_ and *J*_max_ of *G. biloba* less sensitive to salt stress than *R. pulchrum*? This low sensitivity is most likely attributed to small K_leaf_ of *G. biloba* (**Table 3**), that is, only 50% of *R. pulchrum*, resulting a slow transpiration stream that may contribute to less transportation of toxic Na from the soil to the leaves in *G. biloba*. In fact, the leaf Na concentration of *G. biloba* was only 29% of that of *R. pulchrum* under salt stress (**Table 3**). A large interspecific difference in leaf Na accumulation, nearly 30-fold, has been reported for urban trees in Warsaw, Poland (Dmuchowski et al., 2022). In *Z.* sp*ina-christi*, as the Na concentration in the leaves increases, *V*_cmax_ decreases accordingly (Zait et al., 2019). Note that for *G. biloba*, even when Na accumulation is high, salt stress damage remains small. The urban *G. biloba* in Warsaw, Poland, accumulates a remarkable amount of Na, that is, 1061 mg kg^–1^, while no notable damage is observed (Dmuchowski et al., 2019, Dmuchowski et al., 2022).

The low K_leaf_ of *G. biloba* should be affected by two factors: 1) low evaporation rate that is attributed to low stomatal density (**Table 3**) and 2) low hydraulic conductivity in the xylem. The conduit diameter is reported to be important for the response of hydraulic conductivity to soil salinity for angiosperm species, mangroves; species with smaller vessel diameters showed little effect of soil salinity on hydraulic conductivity (Chowdhury et al., 2025). However, the stem tracheid diameter of *G. biloba* is reported to be 17–31µm (Purusatama and Kim, 2020), which is not larger than the stem vessel diameter of *R. pulchrum*, 25 µm (Nilsen, 2009). When we observed the cross section of the petiole of *G. biloba* and *R. pulchrum*, *G. biloba* had a smaller proportion of vascular tissue with lower numbers of tracheids compared to *R. pulchrum* (data not shown). This might be partly related to the low hydraulic conductivity of *G. biloba* (Horike et al., 2023).

### 3.3 Experiment 3: photosynthetic response to long-term excess humidity in *G. biloba*

**Figure 3:**
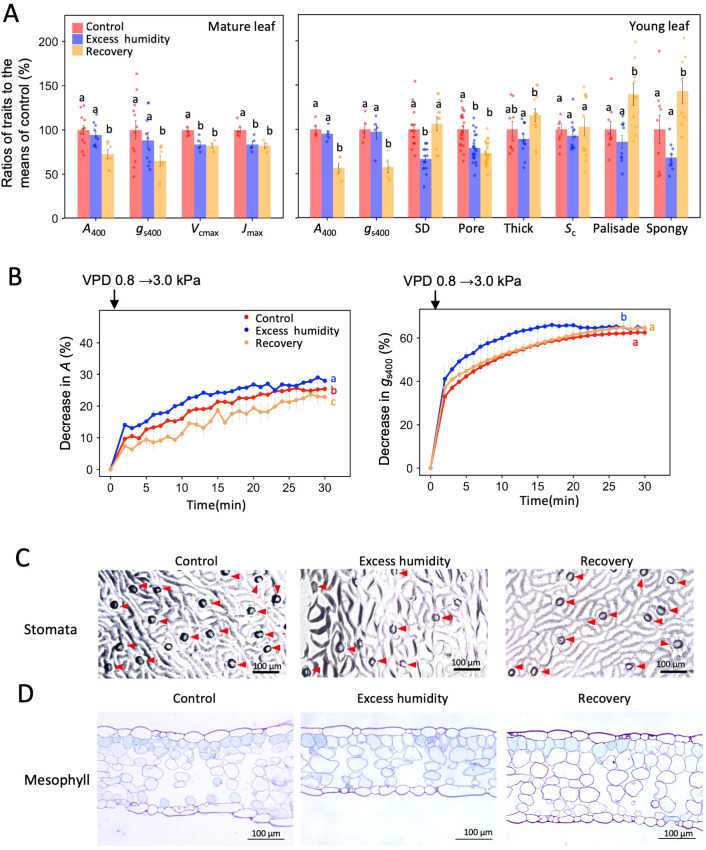
**(A)** Bar plot for the leaf photosynthetic and anatomical traits of *Ginkgo biloba* for mature and young leaves. The ratios of the traits in excess humidity and recovery condition compared to those of the control are shown. Photosynthesis rate (*A*_400_) and stomatal conductance (*g*_s400_, *n* = 13 for mature leaves and *n* = 5 for young leaves), maximum carboxylation rate (*V*_cmax_) and maximum electron transport rate (*J*_max_, *n* = 4–6), stomatal density (SD, *n* = 12–16), stomatal pore length (Pore, *n* = 24), mesophyll thickness (Thick) and surface area of chloroplasts facing the intercellular airspaces (*S*_c_, *n* = 9–12), and palisade cell size and spongy cell size (*n* = 9–10) are shown. **(B)** Time-dependent change in the decrease in *A*_400_ and *g*_s400_ for young leaves when VPD was increased from 0.8 kPa to 3.0 kPa, using the value at VPD of 0.8 kPa as the baseline. ANOVA and Holm’s *post-hoc* test were used to analyze the differences among the treatments in **(A, B)**. Different letters indicate a statistically significant difference. Error bars indicate SE. Light micrographs of **(C)** stomata images from the secondary replica of the lower sides of the leaves, in which red arrows show the stomata and **(D)** the section of the leaves of (*G*) *biloba* that were chemically fixed and embedded in resin.

To assess the response of *Ginkgo biloba* to long-term excess humidity, seedlings of *Ginkgo biloba* were grown under three sequential humidity conditions. The seedlings were grown for two weeks under 1.5 ± 0.2 (mean ± SD) kPa of VPD (control condition), then for three weeks under 0.5 ± 0.2 kPa of VPD (excess humidity condition), and finally for two weeks under 1.6 ± 0.3 kPa of VPD (recovery condition). Excess-humidity-grown plants are expected to show increases in stomatal conductance and photosynthesis due to acclimation of stomata (Nejad and Van Meeteren, 2005). However, when measured at moderate humidity (VPD of 1.3 kPa or 0.8 kPa), the *A*_400_ and *g*_s400_ of the excess-humidity-grown *G. biloba* were no larger and tended to be smaller compared to the control conditions, for both mature and young leaves (**Figure 3A**). This result shows that the stomata of excess-humidity-grown *G. biloba* will no longer be more open when measured at a VPD higher than during growth, even when the difference is small (0.3 - 0.8 kPa). These results suggest sensitive closure of stomata to the increase in VPD.

The sensitive closure of stomata in the excess-humidity-grown *G. biloba* was more apparent in the young leaves when the VPD increased temporarily from 0.8 kPa to 3.0 kPa; a more rapid decrease in *A*_400_ and *g*_s400_ compared to the control plants was observed (**Figure 3B**). These results support previous research showing that *Vicia faba* acclimatized to high humidity conditions had increased stomatal sensitivity to environmental changes, such as changes in the CO_2_ concentration (Talbott et al., 2003). In contrast, a perennial herbaceous plant, *Tradescantia virginiana*, grown under long-term high humidity, showed an increase in *g*_s_ and slow closure of the stomata under low humidity (Nejad and Van Meeteren, 2005). This suggests that the stomatal response to prolonged high humidity is highly species-specific. Such a species-specific response of *g*_s_ may be partly affected by morphological traits of the stomata. The excess-humidity-grown plants showed a significant decrease in stomatal density and stomatal pore length (**Figures 3A, C**), both of which would decrease stomatal conductance (Franks and Beerling, 2009).

Significant reductions in *V*_cmax_ and *J*_max_ in excess-humidity-grown *G. biloba* were observed for mature leaves (**Figure 3A**), which suggests that long-term excess humidity impaired the biochemical functions of *G. biloba* even after leaf maturation. For the *G. biloba* grown under recovery conditions, the significant decline in gas exchange traits, *A*_400_, *g*_s400_, *V*_cmax_, and *J*_max_, were even more pronounced compared to the excess-humidity-grown plants (**Figure 3A**). These results suggest that long-term exposure to excess humidity causes a prolonged negative impact on stomatal function and photosynthetic biochemistry. Even a VPD of 1.6 kPa, which is normally optimal, causes significant stress. On the other hand, the sensitive responses of *A*_400_ and *g*_s400_ to increased VPD observed in plants grown under excess humidity were not so clear under recovery conditions (**Figure 3B**).

What are the effects of mesophyll anatomical changes on the photosynthetic response of *G. biloba* to excess humidity? If the leaf mesophyll tissue of young leaves develops during a treatment of excess-humidity, the surface area of the chloroplasts facing the intercellular spaces (*S*_c_) may increase, which could potentially compensate for the negative impact of biochemical impairment. However, no increase in *S*_c_ was obtained for the excess-humidity-grown plants, nor were there any increases in mesophyll thickness, palisade and spongy cell size (**Figures 3A, D**) or mesophyll cell layers (**Supplementary Table S2**). In contrast, the mesophyll thickness and the sizes of palisade and spongy cells were increased after the recovery treatment (**Figures 3A, D**). These suggests that long-term high humidity inhibited the development of mesophyll in *G. biloba*. The decrease in mesophyll thickness and *S*_c_ due to the inhibition of mesophyll development has been observed in tomato cultivars, but these results were obtained under low humidity, not high humidity (Du et al., 2019). On the other hand, according to Torre et al. (2003), the leaves of roses grown under high humidity become thin, and the numbers of palisade cells and stomatal cells decrease. These suggest that the morphological changes in leaves in response to high humidity are again highly species-specific.

## 4 Conclusions

**Figure 4:**
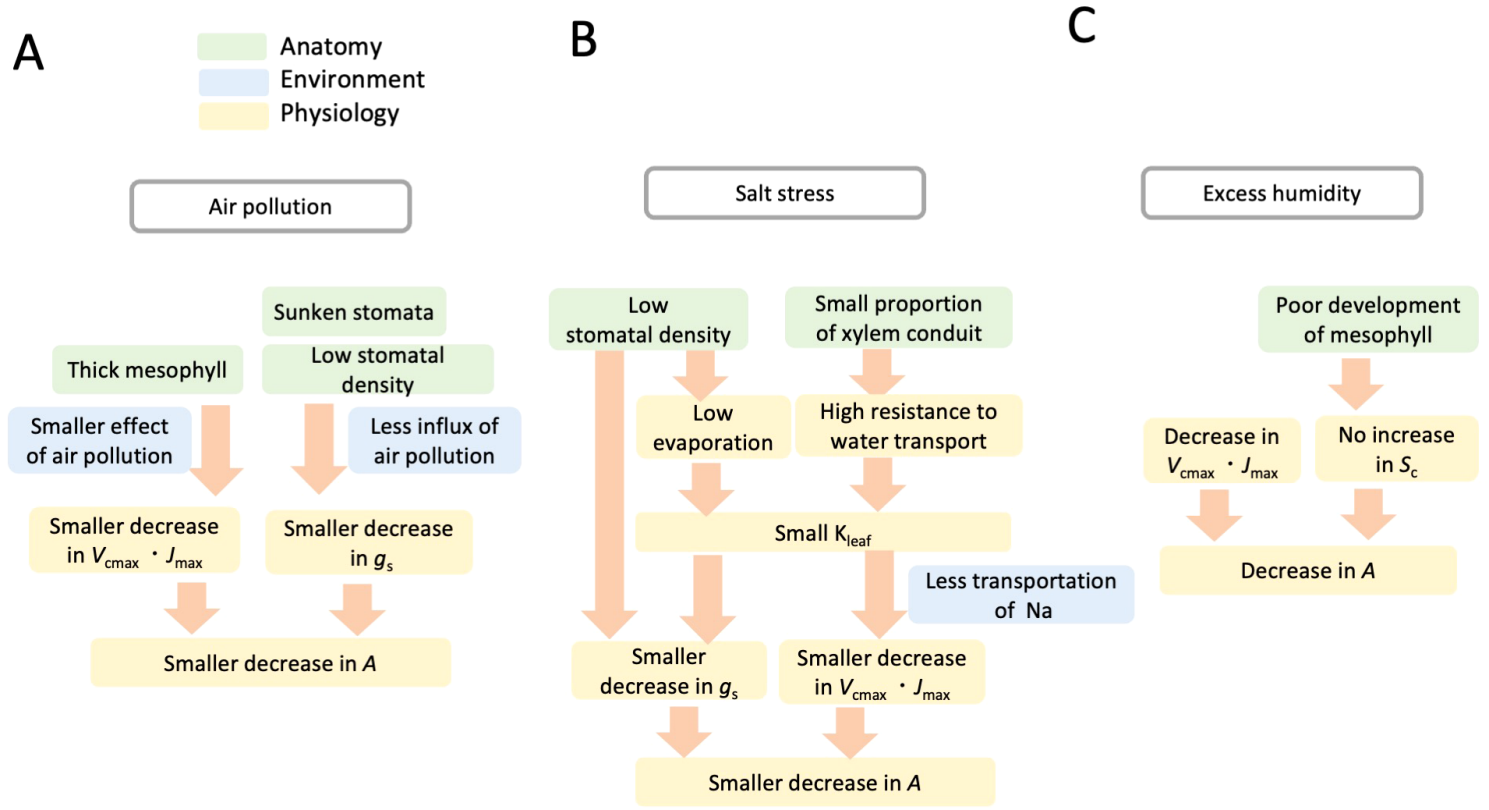
Conceptual diagram of anatomical and physiological mechanisms to explain the photosynthetic response of *Ginkgo biloba* to environmental stresses: air pollution **(A)**, salinity **(B)**, and excess humidity **(C)**. Stomatal, mesophyll, and xylem anatomical traits are key for the photosynthetic response of *Ginkgo biloba*. Explanations are given in the text.

In this study, we focused on three environmental stress factors: (1) air pollution, (2) salt stress, and (3) long-term excess humidity on the photosynthetic response of an urban tree, *G. biloba*. For all of these environmental stresses, the anatomical traits were inferred to be important as a physiological mechanism that explains the response of photosynthetic function in *G. biloba* (**Figure 4**). The lower decrease in *A* in response to air pollution can be attributed to the lower decreases in *V*_cmax_, *J*_max_, and *g*_s_ (**Figure 4A**). Thick mesophyll can mitigate the harmful effects of air pollution due to its high resistance for the diffusion of air pollutants within leaves, which can contribute to a lower decline in photosynthetic biochemistry, *V*_cmax_, *J*_max_. The low stomatal density and sunken stomata from the leaf surface can contribute to a lower influx of air pollutants, which relates to the lower decrease in *g*_s_. For the lower decrease in *A* in response to salt stress, anatomical traits in the leaf, that is, low stomatal density, and xylem anatomical traits, that is, small proportion of xylem conduit to petiole cross section, are possible key traits (**Figure 4B**). Low stomatal density causes a smaller decrease in *g*_s_. Low stomatal density also induces low evaporation, and thus, induces small K_leaf_, resulting in a smaller decrease in *g*_s_. The small proportion of xylem conduit to petiole cross section in *G. biloba* contributes to the high resistance to water transportation, and thus, involves small K_leaf_, which causes less transportation of Na to the leaves. Low accumulation of Na in the leaves causes a smaller decrease in *V*_cmax_ and *J*_max_, which relates in a smaller decrease in photosynthesis. In contrast to the above two environmental stresses, long-term excess humidity imposes a negative impact on the development of leaf mesophyll in *G. biloba*. Excess humidity causes adverse negative effects in photosynthetic biochemistry even after the excess humidity stress is resolved (**Figure 4C**). Therefore, even under favorable humidity conditions of VPD 1.3 kPa, photosynthetic rates remain low.

We demonstrated a high capacity of *G. biloba* to maintain photosynthetic function under stresses such as air pollution and salt stress, both of which exert strong negative effects on urban trees. This confirms the high suitability of *G. biloba* as an urban tree. On the other hand, prolonged exposure to excess humidity caused a sustained decrease in the photosynthetic rate. These results suggest that *G. biloba* does not necessarily possess high tolerance to excess humidity. Future studies are needed to comprehensively elucidate the genetic and physiological mechanisms conferring the high suitability of *G. biloba* as an urban tree by identifying genes involved in these stress tolerance mechanisms.

## Data Availability

The raw data supporting the conclusions of this article will be made available by the authors, without undue reservation.
