# Supplementary material for: Evaluation of the photosynthetic response of Ginkgo biloba as an urban tree to air pollution, soil salinity, and excess humidity
Source: Front Plant Sci. 2026 Feb 11;17:1746328. doi: 10.3389/fpls.2026.1746328 (PMC12932457; doi:10.3389/fpls.2026.1746328)
Supplement: Supplementary file 1 [file Table1.docx]

**Supplementary files**

**Evaluation of the photosynthetic response of Ginkgo biloba as an urban tree to air pollution, soil salinity, and excess humidity**

**Takumi Matsuura^1^, Souma Okugawa^1^, Eri Yamakita^1^, Takashi Kiyomizu^1^, Yuuri Tsutsui^1^, Atsushi Kume^2^ and Yuko T. Hanba^1*^**

^1^ Faculty of Applied Biology, Kyoto Institute of Technology, Matsugasaki, Sakyo-ku, Kyoto 606-8585, Japan

^2^ Faculty of Agriculture, Kyushu University, 6-10-1 Hakozaki, Higashi-ku, Fukuoka 812-8581, Japan

***Correspondence:**Yuko T. Hanba
hanba@kit.ac.jp

**Table S1.** Absolute values of the gas exchange traits and SPAD in the experiment 1.

| Year  Species   Study site (pollution level) | *A*_400_ (µmol m^–2^ s^–1^) | *g*_s400_ (mol m^–2^ s^–1^) | *V*_cmax_ (µmol m^–2^ s^–1^) | *J*_max_ (µmol m^–2^ s^–1^) | SPAD |
| --- | --- | --- | --- | --- | --- |
| 2014 |  |  |  |  |  |
| *Ginkgo biloba* | |  |  |  |  |
| S1 (Low) | 8.13(0.63)a | 0.150(0.022)ab | 41.2(2.3)a | 85.7(3.9)ab | 56.5(1.5)a |
| S2 (High) | 9.12(0.80)a | 0.171(0.030)ab | 57.3(3.7)b | 110.7(3.3)c | 57.1(2.5)a |
| *Rhododendron pulchrum* | |  |  |  |  |
| S1 (Low) | 10.34(1.22)a | 0.252(0.041)a | 48.7(5.2)ab | 90.4(9.4)ac | 39.0(1.5)b |
| S2 (High) | 7.10(0.95)a | 0.128(0.025)b | 36.6(2.3)a | 67.9(5.4)b | 41.8(1.3)b |
| 2017 |  |  |  |  |  |
| *Ginkgo biloba* | |  |  |  |  |
| S3 (Low) | 6.12(0.29)ab | 0.106(0.007)a | 33.0(1.1)ab | 67.9(3.1)a | 47.3(3.7)a |
| S4 (High) | 9.91(0.27)ac | 0.293(0.019)ab | 48.8(1.7)c | 102.3(3.0)b | 58.4(0.7)a |
| *Rhododendron pulchrum* | |  |  |  |  |
| S3 (Low) | 11.80(0.54)c | 0.245(0.024)b | 40.5(2.1)ac | 82.0(4.1)a | 50.3(2.2)b |
| S4 (High) | 6.90(0.35)b | 0.275(0.043)ab | 21.1(2.1)b | 45.6(4.0)c | 39.2(1.9)b |

Means (SE) are shown for *A*_400_ and *g*_s400_ (*n* = 8–16), *V*_cmax_ and *J*_max_ (*n* = 4–15), and SPAD values that reflects chlorophyll content (*n* = 4–15). The effects of the treatment and species were analyzed using ANOVA and Holm’s post-hoc test for 2014 and 2017 separately, where different letters indicate significant difference (p  < 0.05)

**Table S2.** Absolute values of the leaf traits in the experiment 3.

| Traits | Control | Excess humidity | Recovery |
| --- | --- | --- | --- |
| Mature leaves |  |  |  |
| *A*_400_ (µmol m^–2^ s^–1^) | 6.65(0.34)a | 6.26(0.27)a | 4.84(0.32)b |
| *g*_s400_ (mol m^–2^ s^–1^) | 0.163(0.017)a | 0.144(0.014)ab | 0.106(0.010)b |
| *V*_cmax_ (µmol m^–2^ s^–1^) | 34.1(1.1)a | 29.2(0.7)b | 27.1(1.1)b |
| *J*_max_ (µmol m^–2^ s^–1^) | 106.5(3.3)a | 89.0(3.7)b | 87.4(3.1)b |
| Young leaves |  |  |  |
| *A*_400_ (µmol m^–2^ s^–1^) | 7.09(0.29)a | 6.74(0.24)a | 4.01(0.38)b |
| *g*_s400_ (mol m^–2^ s^–1^) | 0.268(0.017)a | 0.260(0.024)a | 0.15(0.016)b |
| Stomatal density (numbers mm^–2^) | 38.8(2.1)a | 25.9(1.4)b | 41.3(3.1)a |
| Stomatal pore length (μm) | 27.9(1.0)a | 22.0(0.9)b | 20.5(0.7)b |
| Mesophyll thickness (μm) | 218(19)ab | 194(11)a | 253(16)b |
| *S*_c_ (m^2^ m^–2^) | 6.96(0.44)a | 6.45(0.34)a | 7.16(0.72)a |
| Palisade cell size (10^3^ μm^2^) | 0.93(0.08)a | 0.79(0.07)a | 1.29(0.11)b |
| Spongy cell size (10^3^ μm^2^) | 1.65(0.27)a | 1.13(0.08)a | 2.76(0.33)b |
| Numbers of mesophyll layers | 6.67(0.41)a | 5.80(0.25)a | 7.00(0.49)a |

Means (SE) are shown for *A*_400_ and *g*_s400_ (*n* =8–12), *V*_cmax_ and *J*_max_ (*n* = 4–6), stomatal density (*n* = 12–16), stomatal pore length (*n* = 24), mesophyll thickness and *S*_c_ (*n* = 9–12), palisade and spongy cell size and numbers of mesophyll layers (*n* = 9–10). The effects of the treatments were analyzed using ANOVA and Holm’s post-hoc test, where different letters indicate significant difference (p  < 0.05)
